# Application of Intrawound Vancomycin Powder during Spine Surgery in a Patient with Dialysis-Dependent Renal Failure

**DOI:** 10.1155/2015/321682

**Published:** 2015-06-22

**Authors:** Jackson Kim, Shane M. Burke, Evan Qu, Steven W. Hwang, Ron I. Riesenburger

**Affiliations:** ^1^Department of Neurosurgery, Tufts Medical Center, Boston, MA 02111, USA; ^2^Department of Neurosurgery, Tufts University School of Medicine, Boston, MA 02111, USA; ^3^Boston University, Boston, MA 02215, USA

## Abstract

Surgical site infections (SSIs) after spinal surgery are a serious complication that can be minimized with prophylaxis. Vancomycin is a common agent used in the prevention of SSI. Given that vancomycin is renally cleared, its use requires careful observation in dialysis-dependent patients due to toxicity at supratherapeutic levels. Since minimum inhibitory concentrations (MICs) for vancomycin have increased due to the emergence of resistant pathogens, the use of vancomycin in such patients is further complicated. Local instillation of vancomycin powder is thought to provide additional protection against SSI and have lower systemic absorption. We present a patient with end-stage renal disease that developed progressively debilitating cervical spondylotic myelopathy necessitating multilevel laminectomy and instrumented fusion. Prior to closure, 1 gram of vancomycin powder was sprinkled into the surgical incision. Postoperative serum vancomycin levels were well below those associated with nephrotoxicity and ototoxicity. Based on this experience, we reviewed the relevant guidelines that were designed to prevent postoperative infections in such dialysis-dependent patients. Intrawound application of vancomycin may be a legitimate and safe option for SSI prophylaxis in patients with renal failure on dialysis.

## 1. Introduction

Surgical site infections (SSIs) are serious complications following spinal surgery, often associated with increased morbidity, mortality, and medical costs [1–7]. Incidence of SSIs after spine surgery has been reported to range between 2% and 15% [4–6, 8–14], with risk factors including dialysis-dependent renal failure [15, 16], posterior approaches [2, 9, 17, 18], revision surgery [19, 20], prior radiation of the surgical field [21, 22], immunosuppression [23], and procedures involving instrumentation [1–3, 9, 17, 18, 24]. SSI is also more likely in diabetic patients, with studies suggesting a 2- to 5-fold increased risk over nondiabetic patients [7, 25, 26].

Since the majority of SSIs involve Gram-positive bacteria [27, 28], vancomycin is a common component in prophylactic antibiotic therapy [29, 30]. Vancomycin, however, needs to be used judiciously as it can also be nephrotoxic, ototoxic, and myelosuppressive at high levels, a significant concern given increasing recommended serum trough levels (15–20 ug/mL) when administered intravenously [29–43]. Increasing rates of vancomycin-related side effects have been reported in the literature, presumably due to the aggressive doses needed to achieve an appropriate minimum inhibitory concentration (MIC) [32, 33, 39, 41, 43–45].

Given that vancomycin is renally cleared, its use requires careful observation in patients with renal failure due to toxicity at supratherapeutic levels. Since MICs for vancomycin have increased due to more virulent forms of methicillin-resistant* Staphylococcus aureus* (MRSA), the use of intravenous vancomycin in such patients is further complicated. Intrawound application of vancomycin powder is an increasingly used route of antibiotic administration that is a potentially attractive alternative to the intravenous route. Recent literature suggests that local intrawound application of vancomycin powder provides protection against SSI [4]. It also may carry less side effects as the systemic absorption of intrawound vancomycin appears to be low. To our knowledge, the use of intrawound vancomycin powder has not been reported in the literature in a dialysis-dependent patient, possibly because of the concern that supratherapeutic levels may precipitate unacceptable morbidity. We present the first reported case of the safe application of intrawound vancomycin prophylaxis in a patient with end-stage renal disease on hemodialysis, without a significant elevation of serum vancomycin levels.

## 2. Case Presentation

A 72-year-old man was followed up in our clinic for two years for early cervical myelopathy secondary to C3 to C5 stenosis. His medical history was significant for dialysis-dependent end-stage renal disease due to chronic allograft nephropathy despite cyclosporine therapy for a living-related donor kidney transplant. He suffered a previous myocardial infarction treated with percutaneous coronary intervention and sick sinus syndrome, which required a pacemaker that was removed due to a postoperative abscess. He survived squamous cell carcinoma of the parotid gland with lymphatic involvement that necessitated radical resection and radiation of the head, neck, and axilla. He was also diabetic. At the time of his initial presentation to our clinic, his symptoms included very slight loss of coordination in his left hand and a mild sense of imbalance. He could still ambulate and live independently and therefore did not wish to proceed with cervical decompression and fusion.

He was lost to follow-up but returned to our clinic two years later. At this time, he presented with loss of coordination in both hands and could no longer ambulate. As a result, he was no longer able to care for himself. He remained dialysis dependent and was oliguric, but not completely anuric. Repeat cervical MRI revealed worsening stenosis at C3-5 compared to the MRI scan performed two years earlier. At this point, he wished to proceed with surgery. As per standard protocol, he was given 1 gram of intravenous cefazolin immediately prior to incision. He underwent posterior C3-5 laminectomies and instrumented fusion with lateral mass fixation. In an attempt to preserve his remaining kidney function and reduce his risk of SSI, we applied 1 gram of vancomycin powder over the instrumentation and soft tissues of the surgical exposure. Although there were not any previous reports of intrawound application of vancomycin powder in patients on dialysis, we felt it was a justified treatment to minimize his chance of SSI given his multiple risk factors for a postoperative infection.

A serum vancomycin level six hours after surgery was 0.5 *μ*g/mL. The level remained at 0.5 *μ*g/mL in the morning following surgery. By postoperative day two, the vancomycin level was less than 0.2 *μ*g/mL. There were no postoperative complications or changes in the patient's dialysis regimen. The surgical wound was well healed at six weeks' follow-up. It remained well healed without signs of infection 10 months later.

## 3. Discussion

We present a dialysis-dependent patient with multiple risk factors for SSI in whom intraoperative administration of vancomycin powder was used without further worsening of his remaining kidney function and without any other vancomycin-related side effects. Several previously reported risk factors for SSI were applicable to this patient: dialysis-dependent renal failure [15, 16], a posterior approach [2, 9, 17, 18], previous neck radiation [21, 22], immunosuppression to prevent kidney transplant rejection [23], diabetes [9, 12, 15, 17, 24, 25, 46], and a recent history of pacemaker explantation secondary to a postoperative abscess. While vancomycin prophylaxis may be advised in patients with such comorbidities [47], the potential side effects of intravenous vancomycin present a clinical challenge in patients that have both a high risk of SSI and low renal reserve [29, 30, 33–37]. This case supports intrawound application of vancomycin powder as a potential solution for these challenging patients.

Current guidelines for patients with comorbidities who receive instrumented fusion recommend further prophylaxis in addition to the standard single dose of a preoperative intravenous antibiotic with intraoperative redosing [47]. This can include Gram-negative coverage and/or intrawound vancomycin or gentamicin [47]. While the guidelines state there is insufficient evidence to recommend either of these regimens over the other [28, 47], intrawound antibiotics have been shown to significantly reduce the incidence of SSI after instrumented fusions [4, 6, 7, 9, 48, 49]. Therefore, we selected intrawound vancomycin in this patient due to his compromised kidney function.

The treatment of patients on hemodialysis with intravenous vancomycin is complicated. Not only are accepted dosing normograms for patients with ESRD becoming outdated [30, 50, 51], but the intermittent nature of the hemodialysis makes stable levels difficult to achieve [30, 50]. One study found that only 57.3% of hemodialysis patients reached the recommended MIC for vancomycin, even with postdialysis administration of the drug [52]. Yet another study found that serum vancomycin levels rebounded after hemodialysis to treat vancomycin toxicity [38, 53]. This paradoxical response suggests protein binding that buffers shifts of free serum vancomycin levels in any extreme direction [38].

Intrawound drug instillation theoretically avoids this effect due to poor tissue penetration [6, 54–56]. Vancomycin is reported to only have 10–30% absorption from skin and soft tissue (in both normal and diabetic patients) [29, 30]. This absorption may be further limited by local tissue ischemia, seroma, and hematoma [9]. Such factors are thought to facilitate high local concentrations without increasing serum levels [6, 54–57]. One study found the tissue antibiotic level to be 1000 times greater than the MIC for MRSA and coagulase negative* Staphylococcus aureus* with intrawound administration [6]. Some have even suggested that intrawound antibiotics alone (i.e., without any intravenous antibiotics) are adequate to reduce the risk of postoperative SSIs [48].

While the preceding evidence implies that local vancomycin is effective in SSI prophylaxis and can avoid serum fluctuations, its efficacy has never been assessed in relation to nephrotoxicity in patients on dialysis. The most relevant data on this topic has been investigated in otherwise healthy patients. In this retrospective cohort analysis, mean serum vancomycin levels trended downward from 2.5 *μ*g/mL over the first two postoperative days after intraoperative vancomycin powder instillation. The study further demonstrated that mean drain concentrations (a marker of local concentrations) were over 100 *μ*g/mL at 2 days after surgery, which suggests that local therapeutic levels of vancomycin were maintained.

While the limited toxicity afforded by local instillation of vancomycin is attractive, such therapy is not without risks. Some studies have demonstrated that nonselective use of local antibiotic monotherapy could lead to resistance [49, 58]. Preventing resistance is especially important in scenarios where a nidus for infection exists, such as in the case of instrumented fusions [2], since multidrug resistant postoperative hardware infections can lead to serious complications. In addition, some animal studies have even shown that intrawound vancomycin can inhibit osteoblasts [9]. In human osteosarcoma cell line, however, fusion rates have not been affected with tissue levels below cytotoxic levels [6, 9]. Finally, hemodynamic collapse has been reported with the use of intrawound vancomycin in patients with underlying heart disease [59]. Therefore, given the potential risks of therapy, moderation must still be employed to limit this route of vancomycin to those patients who have medical factors to indicate its use.

## 4. Conclusion

In summary, this case represents the first reported case of the safe application of intrawound vancomycin prophylaxis in a patient with end-stage renal disease on hemodialysis, without reaching supratherapeutic levels. In doing so, this case supports the premise that poor soft tissue absorption of vancomycin powder limits serum levels. As serious comorbidities and multidrug resistant infections become more prevalent, SSI prophylaxis must similarly advance to address these challenges. This report suggests that intrawound vancomycin may be a viable option for SSI prophylaxis in patients with ESRD on dialysis. Thus, this and other local therapies may prove to be a central theme of future research.
